# Temporal Dynamics of User Engagement in Professional Video Communities: A Time-Series Clustering Analysis Based on Bilibili’s Legal Content

**DOI:** 10.3390/e28060651

**Published:** 2026-06-09

**Authors:** Chuchu Liu, Haorun Li, Shuyang Zhao, Xiaoqing Zeng, Xin Lu

**Affiliations:** 1 School of Economics and Management, Changsha University of Science and Technology, Changsha 410076, China; 2 College of Systems Engineering, National University of Defense Technology, Changsha 410073, China

**Keywords:** video community, comment evolution, pattern discovery, time-series clustering, user engagement

## Abstract

Presently, video communities such as YouTube, bilibili and TikTok have emerged as core fields for information dissemination and public opinion generation. Their embedded user dynamic interaction data support research on public cognitive behavior and content dissemination laws. This study used web crawling technology to construct a complete dataset including 367 video metadata and 2.39 million comment records from *Luo Xiang Speaks on Criminal Law*—a prominent legal popularization account on the bilibili platform—and systematically explored the temporal evolution patterns of comment interactions in video communities. By establishing a four-dimensional feature system alongside the k-means++ clustering algorithm, this study successfully identified three distinct comment growth patterns (*p* < 0.001): the burst–decay, the multi-wave oscillation, and the delayed peak. The results of non-parametric tests showed that these three patterns have significant differences in core features (e.g., peak delay time, skewness) and are systematically related to user grade structure, content interaction depth, and release timing. In addition, the user interaction networks of different videos demonstrate significant structural heterogeneity and disassortative mixing, characterized by a highly active minority dominating the discourse, while peripheral nodes gravitate toward high-profile hubs. These findings offer researchers deeper insights into the micro-mechanisms of information dissemination.

## 1. Introduction

Video platforms such as bilibili and YouTube, leveraging interactive features like bullet comments, comments, and sharing, foster highly active community ecosystems that facilitate real-time interaction and content co-creation. The massive user-generated content (UGC) accumulated on these platforms directly mirrors the distribution of public attention, the evolution of social issues, and cultural trends. Furthermore, the inherent time-series structure of this data provides a robust empirical foundation for characterizing group-level interaction dynamics and participation patterns. Notably, user comment growth within these video communities exhibits significant heterogeneity. The interaction patterns of different videos often have distinct differences, e.g., some videos’ comments show a short-term burst followed by a rapid decline, while others exhibit periodic growth with multiple fluctuations. This differentiated interaction pattern is of significant research value for in-depth understanding of user participation mechanisms, evaluating content dissemination effectiveness, and exploring the sustainable vitality of the community. By collecting and analyzing authentic user comments in video communities, this study aims to systematically explore the following 3 questions. Does user comment behavior exhibit typical patterns over time? What are the dynamic characteristics of these patterns? And what are the differences in the underlying formation mechanisms?

Bilibili, often affectionately known as “B Zhan” (B Site), is one of China’s most influential video-sharing and social media platforms [1]. Today, bilibili serves as a primary hub for China’s Gen Z and Gen Alpha, driven largely by professional user-generated videos created by talented “UP masters”. Notably, it is not just a video site, but a vibrant social community where creators and viewers foster deep connections through shared passions. And the comment section on bilibili is a public discourse space for viewpoint exchange and knowledge reproduction, providing a high-density and structurally complete data foundation for observing user engagement behaviors. Specifically, *Luo Xiang Speaks on Criminal Law* as the most influential legal knowledge IP on bilibili, with its professional content, real-world relevance, and social discussion value, has attracted a large number of users for frequent interaction and diverse viewpoints collisions. The comment section of this IP reflects the complexity of user engagement behaviors in legal knowledge-related videos, making it a typical sample for exploring the evolution patterns of video comments and user participation behaviors.

Bilibili’s platform architecture shapes a unique comment evolution mechanism that departs significantly from mainstream social media. First, its focus on mid-to-long-form videos provides the cognitive runway necessary for reflective, substantive discourse. Second, the danmaku system introduces a powerful diversion effect: by absorbing real-time affective reactions during video playback, it alleviates the pressure for instant gratification in the traditional comment section, fostering highly non-linear and delayed evolution patterns in textual comments. Third, a strict, formalized user-leveling system instantiates a highly hierarchical interaction structure, allowing a dedicated core user base to govern the discursive flow. Together, these architectural affordances anchor a deep discursive ecology rich in textual analysis, meme cultural symbols, and knowledge production—a stark counterpoint to the brief, reactive interaction patterns observed on short-form video platforms. Consequently, comment trajectories on Bilibili manifest unprecedented non-linearity and localized cultural specificity.

Current studies have explored the bilibili platform from multiple dimensions, preliminarily identifying its platform characteristics, user behaviors, and content dissemination patterns [2,3,4]. Based on over 2 million videos and 28 million user data, Jia et al. [5] achieved real-time prediction of video popularity by constructing a graph model that integrates user attention relationships and comment interactions. Rong et al. [6] further defined bilibili as a two-sided platform, and through comparative analysis, pointed out that its user stickiness mainly stems from network effects and proprietary resources. In terms of content quality assessment, Zheng et al. [7] found that content released by professional institutions was significantly more rigorous. However, the overall quality of medical information on the platform still requires improvement. Xu et al. [8] identified high-activity segments from the bullet comments of travel vlogs, decomposed viewer participation into narrative participation, quasi-social interaction, etc., and constructed a framework for the association between content types and travel intentions. Ojer et al. [9] developed a dynamic model of individual attention for formal mechanistic description, suggesting that user attention in online social interactions declines exponentially over time. Firth et al. [10] argued that online social engagement and information consumption are dominated by fleeting attention. People’s interest in certain topics or cultural products naturally diminishes over time, with the half-life reduced to days or even hours. Nguyen et al. [11] stated that short videos’ endless scrolling and algorithm-powered instant gratification create a fragmented, high-frequency information consumption pattern.

Overall, current studies mainly focuses on popularity prediction [12], platform business models [6] content quality assessment [13], and analysis of user interaction behaviors [14] on bilibili, with insufficient attention paid to the dynamic patterns of user comment behaviors. Moreover, there remains a lack of systematic exploration regarding the typical evolutionary patterns of comment growth curves.

To capture how comment engagement evolves over time, this study attempted to employ time-series clustering methods to identify similar structural patterns in user comments. Time-series clustering categorizes sequences through feature extraction and similarity measurement, encompassing four primary paradigms: shape-based, model-based, machine learning-based, and feature-based [15,16,17]. Shape-based methods, such as Dynamic Time Warping (DTW) [18,19,20] and the K-shape algorithm [21,22], directly assess similarity based on raw sequence morphology. While capable of handling temporal shifts, these approaches often incur high computational complexity and are sensitive to amplitude scaling, limiting their effectiveness for highly sparse and bursty social media comment data. Model-based methods, such as Hidden Markov Models (HMM) [23] and Gaussian Mixture Models (GMM) [24], assume that data follows specific probability distributions or generative processes, achieving clustering via parameter estimation. However, these methods rely on strong prior assumptions and exhibit limited fitting capabilities for non-stationary, non-linear comment data; furthermore, they are susceptible to outliers and local optima [25]. Deep learning-based methods [26], such as LSTMs [27], Transformers [28], and CNNs [29], utilize deep neural networks for representation learning and cluster assignment, enabling the capture of complex non-linear temporal dependencies. However, these methods typically necessitate large-scale labeled datasets, incur substantial computational costs, and suffer from limited interpretability. Furthermore, they often exhibit inadequate robustness when dealing with sparse and noisy data [30,31]. By contrast, feature-based clustering methods [32,33,34] transform high-dimensional time-series data into low-dimensional feature representations through feature engineering, then perform clustering analysis. These methods do not rely on rigid generative assumptions or extensive labeled datasets. Instead, they effectively mitigate observational noise and accommodate variable-length sequences [35,36]. Furthermore, by identifying interpretable features, such as trends, fluctuations, and peaks, these approaches are particularly well-suited for addressing the high sparsity, burstiness, and non-stationarity inherent in video comment data [37,38]. Therefore, this study employs a feature-based time-series clustering framework. Utilizing full-cycle video and comment data from the bilibili channel *Luo Xiang Speaks on Criminal*, we identify the typical patterns of comment popularity evolution. Additionally, statistical tests are conducted to verify significant differences among these patterns, thereby revealing the general regularities of video activity cycles and their underlying formation mechanisms.

## 2. Data and Methods

### 2.1. Data Source

This study collected the complete public data of the well-known legal education account *Luo Xiang Speaks on Criminal Law* on bilibili with a web crawler to explore the temporal evolution pattern of user comment behavior [39]. The basic information of all videos published from the account’s establishment until 31 August 2024 was collected, including video ID, release time, view count, etc. Further, we employed a hierarchical collection strategy to systematically obtain the comment and reply data in the comment sections of all videos. In the end, a full dataset (Table 1) covering 367 video records and 2.39 million user comments was built to support subsequent temporal analysis.

### 2.2. Data Preprocessing

To ensure data quality, this study first pre-processed the collected dataset by excluding comment records with missing critical fields, such as timestamps and user IDs. Since advertisements can severely interfere with subsequent clustering analysis and introduce data noise, we constructed a promotional keyword lexicon (e.g., “Add WeChat”, “Click the link”) to filter them out. Subsequently, the comments for each video were aggregated on an hourly basis to construct a time series set $D=\{X_{n}{\}}_{n=1}^{N}$, where $X_{n}=\{x_{nt}{\}}_{t=1}^{T}$ represents the sequence of comment counts for the n-th video within T time units, and $x_{nt}\in N$ (natural numbers) is the number of comments for the n-th video in the t-th hour. Analysis revealed that for the majority of videos, comment counts ceased to grow or grew slowly after the fourth day post-release. Therefore, the original sequences of each video (with a length of $L_{i}$) were truncated or zero-padded, and the length of all time series was uniformly controlled to 4 days (96 h),

(1) $${\tilde{X}}_{n}=\left\{\begin{array}{llll} X_{n}\left[0:96\right] & L_{n}\geq 96 & & \\ pad\left(X_{n},96-L_{n}\right) & L_{n}\leq 96 & & \end{array}\right.$$

where pad(⋅) is the zero-padding function, used to fill the end of the original sequence $X_{n}$ with zero to make its length reach 96; $X_{n}\left[0:96\right]$ represents the first 96 elements of the original sequence. Finally, Z-score standardization was carried out to eliminate the influence of units,

(2) $$z_{n}\left(t\right)=\frac{{\tilde{x}}_{n}\left(t\right)-\mu_{n}}{\sigma_{n}}$$

where $\mu_{n}$ and $\sigma_{n}$ represent the mean and standard deviation of the i-th sequence.

### 2.3. Feature Construction

Before performing clustering, this study implemented a feature construction and selection process to maximize discriminative power while minimizing redundancy. The justification for our feature selection lies in four observed data characteristics: (i) user comments are typically concentrated within specific intervals (e.g., immediately following a video’s release); (ii) interaction behaviors exhibit diel periodicity; (iii) key users act as the core drivers of engagement; and (iv) the majority of users interact sporadically rather than sustainedly. Building upon these features, we divided the time series features into four categories: trend features, peak features, extreme value features, and statistical features, to prepare for the subsequent time series clustering. The selection of these four categories is theoretically motivated by well-established findings on human dynamics and social contagion [40]. Trend features (rise ratio, fall ratio, turning points, max–min sum) are chosen to capture the bursty nature of human interactions [41,42]. Peak features (number of significant peaks, peak interval) are included to detect rhythmically recurrent engagement windows, grounded in the empirical regularity that human behaviors possess strong periodicities and high predictability [43]. Extreme-value features (maximum index) target the exact temporal loci of hub-driven amplification, reflecting the finding that a small minority of influential members disproportionately drive information diffusion in social networks [44]. Statistical features (skewness, kurtosis) are employed to characterize the distributional signatures of sporadic participation and heavy-tailed engagement, consistent with the observation that public discourse formation is dominated by a highly active few while the majority participate only sporadically [45].

(1) Trend features

This study described the trend characteristics using the first-order difference sequence of the time series, including the rise ratio (Rise_Ratio), the fall ratio (Fall_Ratio), turning points (Turning_Points), and the sum of max–min differences (Max_Min_Sum). These four features can effectively identify the increasing and decreasing trends, fluctuation situations, and variation amplitudes of the sequence, reflecting the trend characteristics of the time series. The rise ratio (Rise_Ratio) and the fall ratio (Fall_Ratio) calculate the proportions of the rising and falling segments within the entire sequence, respectively,

(3) $$Rise\_Ratio=\frac{1}{L_{n}-1}\sum_{i=1}^{L_{n}-1}\delta \left(\Delta x_{i}>0\right)$$

(4) $$Fall\_Ratio=\frac{1}{L_{n}-1}\sum_{i=1}^{L_{n}-1}\delta \left(\Delta x_{i}<0\right)$$

where $\Delta {x_{i}=x}_{i+1}-x_{i}$, $\delta \left(\cdot \right)$ is an indicator function. The rise ratio and the fall ratio capture the changing direction of the time series and can effectively distinguish the increasing and decreasing trends.

Turning points (Turning_Points) refer to the number of times that the first-order difference changes from positive to negative or from negative to positive, which represents the number of times an upward trend turns into a downward trend or vice versa,

(5) $$sign\left(\Delta x_{i}\right)=\left\{\begin{array}{llll} 1 & & & \;\Delta x_{i}>0\\ 0 & & & \;\Delta x_{i}=0\\ -1 & & & \;\Delta x_{i}<0 \end{array}\right.$$

(6) $$\delta_{i}=\left\{\begin{array}{ll} 1 & if\;sign\left(\Delta x_{i}\right)\neq sign\left(\Delta x_{i+1}\right)\;\\ 0 & \;otherwise \end{array}\right.$$

(7) $$Turning\_Points=\sum_{i=1}^{n-2}\delta_{i}$$

where $\delta_{i}$ determines whether the point corresponding to the i-th time step is a turning point. If $\delta_{i}=1$, it indicates that it is a turning point; otherwise, it is not. Turning points are insensitive to the amplitude scaling of the sequence, and can accurately quantify the change frequency of the time series.

The sum of max–min differences (Max_Min_Sum) represents the sum of the maximum and minimum values in the first-order difference. Its sensitivity to extreme values allows it to accurately characterize drastic fluctuations within the sequence,

(8) $$Max\_Min\_Sum=max\left(\Delta x_{i}\right)+min\left(\Delta x_{i}\right)$$

(2) Peak features

In time series analysis, peaks, serving as turning points in rising or falling trends and representing local maxima, often encapsulate the core characteristics of a sequence through their position, magnitude, and frequency. However, video comment time series are characterized by a high temporal concentration of comment volume interspersed with sporadic, minor fluctuations; consequently, relying on traditional methods of identifying extreme points to characterize peaks often generates a multitude of spurious extrema, thereby obscuring the true peak features. To accurately identify peaks with analytical value, this study conducted a comprehensive discrimination from three core dimensions: peak magnitude, inter-peak distance, and local prominence. This mechanism constructs a dynamic threshold adaptive adjustment strategy by integrating the sequence dynamic range and standard deviation compensation, simultaneously setting triple constraint thresholds for peak magnitude, inter-peak distance, and local prominence, and introducing a double-boundary protection mechanism to achieve precise identification of significant peaks and noise filtering.

The significance of the peak magnitude is determined by simultaneously constraining the sequence dynamic range and the degree of fluctuation. Firstly, the base threshold is calculated,

(9) $$H_{base}=\alpha *R+\beta *\sigma$$

where $R=\max\left(x\right)-min(x)$ represents the range of the sequence; σ is the standard deviation; α and β are parameters (set to 0.06). To prevent missed detections caused by excessively high thresholds in sequences with high noise, or false detections resulting from excessively low thresholds in stable sequences, a dual-boundary protection mechanism is further introduced,

(10) $$H_{final}=max\left(\gamma_{min}R,min\left(\gamma_{max}R,H_{base}\right)\right)$$

where $\gamma_{min}=0.03$ and $\gamma_{max}=0.13$. This ensures that the threshold maintains its discriminatory power even under extreme conditions.

To constrain the inter-peak distance and prevent multiple extreme values from being identified within a small range, an adaptive peak spacing constraint is introduced,

(11) $$D_{min}=max\left(\delta_{min},(n/\eta )\right)$$

where $\delta_{min}=2$ denotes the minimum distance constant, n represents the sequence length; and η=48 serves as a tuning parameter enabling the spacing threshold to adapt dynamically to the sequence length.

To further validate the local prominence, prominence P is introduced to measure the significance of a peak relative to its surrounding troughs,

(12) $$P=H_{peak}-max\{V_{left},V_{right}\}$$

where $H_{peak}$ is the height of the peak, $V_{left}$ is the height of the lowest trough on the left side of the peak, and $V_{right}$ is the height of lowest trough on the right side. A peak is considered prominent if its prominence P is greater than $P_{min}$,

(13) $$P_{min}=0.5*H_{final}$$

The number of significant peaks (Num_Significant_Peaks) refers to the count of local maximum points that simultaneously satisfy the above three constraints, which are calculated by the method mentioned earlier,

(14) $$N_{\mathrm{significant}}=\sum_{i=1}^{M}I\left(x_{i}>H_{final}\wedge \Delta t_{i}\geq D_{min}\wedge P_{i}>P_{min}\right)$$

where M represents the total number of candidate peaks, $x_{i}$ denotes the amplitude of the point, $\Delta t_{i}=t_{i+1}-t_{i}$ represents the distance between the i-th and (i+1)-th peaks, and I(⋅) denotes the indicator function, whose mathematical definition is given as follows: I(⋅)=1 if the condition inside the parentheses holds; otherwise, I(⋅)=0.

The mean interval of significant peaks (Peak_Interval_Mean) is used to measure the average distance between adjacent peaks in a time series, reflecting the distributional compactness of concentrated interactions,

(15) $$Peak\_Interval\_Mean=\left\{\begin{array}{lll} \frac{1}{N-1}\sum_{i=1}^{N-1}\Delta t_{i}, & N\geq 2 & \\ 0, & N<2 & \end{array}\right.$$

where N represents the number of significant peaks, and $\Delta t_{i}=t_{i+1}-t_{i}$ indicates the distance between the i-th and (i+1)-th peaks. The significant peak interval serves as an indicator of the density of concentrated interactions within video comments. A narrow spacing suggests that concentrated interactions are dense, signifying that user attention toward the topic is focused and sustained. Conversely, a wide spacing indicates that concentrated interactions are dispersed, triggered intermittently rather than constituting a continuous, heated discussion.

(3) Extreme value features

Analysis of video comment sequences reveals a characteristic of a high concentration; consequently, the position at which the maximum value occurs is directly linked to the core patterns of comment growth. Therefore, this study separated the feature of maximum values from the broader framework of extreme values, focusing specifically on the location of the maximum to provide a more targeted discriminative basis for time series clustering. The maximum value index (Max_Index) indicates the temporal position at which the global maximum value occurs within a time series, serving to pinpoint the specific time point when a video was most widely discussed.

(16) $$Max\_Index=t_{max}-t_{0}$$

where $t_{0}$ marks the beginning index of the time series, and $t_{max}$ indicates the exact time index at which the global maximum occurs.

(4) Statistical features

The distribution symmetry and steepness of time series play a crucial role in identifying time series patterns. In this study, skewness and kurtosis are selected to describe the distribution characteristics of time series. *Skewness* ($g_{1}$) is an indicator commonly used to measure the asymmetry of data distribution,

(17) $$g_{1}=\frac{1}{L_{n}}\sum_{i=1}^{L_{n}}x_{i}^{3}$$

where $L_{n}$ denotes the length of the time series, and $x_{i}$ represents the standardized value of the number of comments in the i-th time unit.

*Kurtosis* ($g_{2}$) quantifies the peakedness and tailedness of a probability distribution,

(18) $$g_{2}=\frac{1}{L_{n}}\sum_{i=1}^{L_{n}}x_{i}^{4}-3$$

where $L_{n}$ denotes the length of the time series, and $x_{i}$ represents the standardized value of the number of comments in the i-th time unit. In this study, a total of 9 features covering trend, peak, extreme value, and statistical characteristics were constructed, as shown in Table 2.

(5) Feature selection based on multi-criteria scoring

In order to remove noise and redundant information, and improve the clustering performance, multi-criteria feature scoring was adopted to screen constructed features. Feature importance is determined using a multi-criteria scoring system that integrates Principal Component Analysis (PCA), autocorrelation intensity, and clustering stability, with weights assigned at 0.6, 0.25, and 0.15, respectively. Specifically, the PCA component (0.6) prioritizes essential discriminatory information regarding video comment growth patterns while minimizing interference from secondary features. The autocorrelation weight (0.25) identifies features sensitive to temporal dependencies without over-amplifying transient noise. Finally, clustering stability (0.15) is incorporated to enhance model generalization and prevent overfitting.

**Figure 1:**
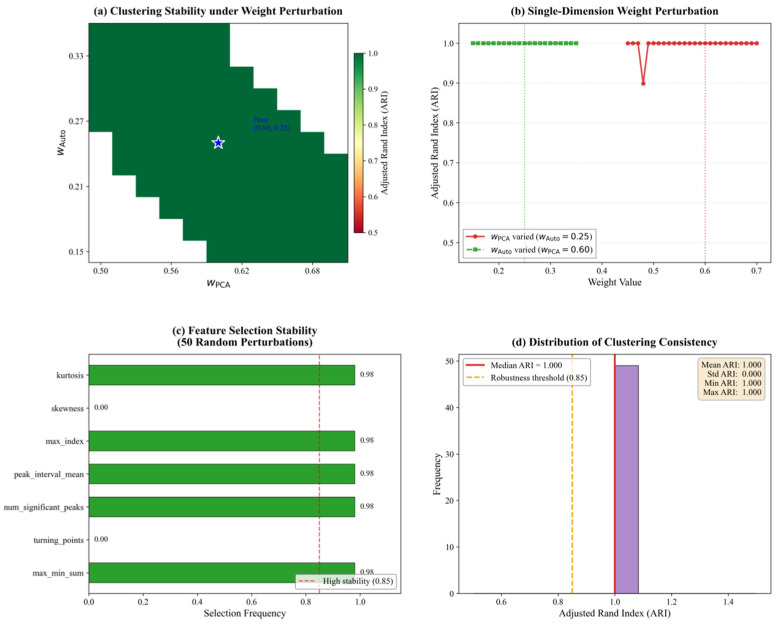
Robustness validation of multi-criteria feature weighting. (**a**) Clustering stability under two-dimensional weight perturbation; (**b**) Single-dimension weight perturbation trajectories. The dotted lines in the figure represent the parameter values selected for this study; (**c**) Feature selection stability across 50 random perturbations; (**d**) Distribution of clustering consistency between perturbed and baseline labels.

To verify the robustness of the feature weighting configuration, we systematically perturbed the weights through four extensive tests (Figure 1). First, a two-dimensional grid scan ($W_{PCA}$ ∈ [0.50, 0.70], $W_{Auto}$ ∈ [0.15, 0.35]) evaluated clustering consistency via the Adjusted Rand Index (ARI). The ARI maintained a stable plateau (≈1.0) across $W_{PCA}$ ∈ [0.56, 0.70] and $W_{Auto}$ ∈ [0.15, 0.33], confirming that our baseline configuration (0.60, 0.25) resides securely within a high-robustness region. Second, single-dimension perturbation tests, fixing $W_{Auto}$ = 0.25 while varying $W_{PCA}\;$ (or fixing $W_{PCA}$ = 0.60 while varying $W_{Auto}$), induced no significant drop in ARI (a negligible dip to 0.90 occurred only at an extreme boundary), indicating minimal marginal effects. Third, feature-selection stability was validated using 50 Monte Carlo random perturbations. Five core features (*Kurtosis*, *Max_Index*, *Peak_Interval_Mean*, *Num_Significant_Peaks*, and *Max_Min_Sum*) exhibited a selection frequency of 0.98, effectively isolating them from unstable, filtered-out variables. Fourth, across all 50 perturbations, the ARI between the perturbed clustering labels and the baseline remained perfectly consistent at 1.000 (with a standard deviation of 0.000), substantially exceeding the pre-established robustness threshold of 0.85. Collectively, these findings demonstrate that our weight assignment occupies a broad, stable plateau, rendering the core feature subset immune to perturbation and ensuring highly reproducible clustering outcomes.

The feature selection process is initialized with the following parameters: minimum feature count $k_{min}=5$, step size L=2, and convergence threshold M=0.01. The number of clusters c is empirically set between 2 and 8. Starting from $k=k_{min}$, feature subsets are iteratively constructed and evaluated using k-means++ clustering. The process monitors the maximum Silhouette Coefficient S(k) and terminates when the convergence criterion |S(k)−S(k−L)|<M is met, yielding an initial optimal count $k_{current}$. Subsequently, a local fine search is performed within the interval $\left[k_{current}-L+1,k_{current}+L-1\right]$ to determine the final optimal number $k_{fin}$. This dual-stage search ensures that the selected subset captures core discriminatory information while minimizing computational complexity, thereby enhancing the accuracy of subsequent time-series clustering. Consequently, the proposed selection mechanism identified seven optimal features, including Turning_Points, Skewness, Kurtosis, Max_Min_Sum, Num_Significant_Peaks, Max_Index, and Peak_Interval_Mean.

### 2.4. Methodological Framework

To address the high sparsity, burstiness, and non-stationarity inherent in video comment time series, this study employed feature-based clustering. This method mitigates observation noise and accommodates unequal sequence lengths by extracting interpretable features such as trends, peaks, and statistical distributions. Using this approach, we analyzed 367 videos from the *Luo Xiang Speaks on Criminal Law* account to identify and classify temporal patterns of comment popularity, thereby revealing the dynamics of user engagement within the video community.

**Figure 2:**
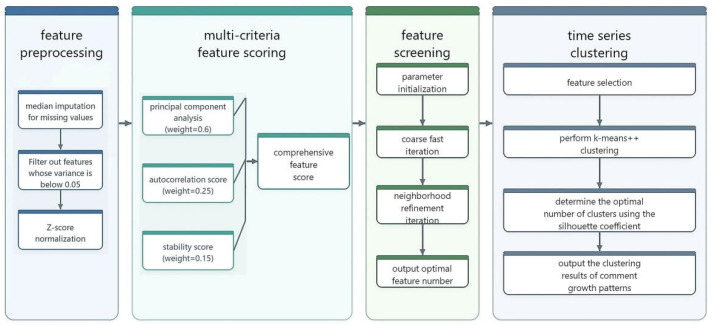
Flowchart of the clustering process.

Details are shown in Figure 2. Initially, 9 features were extracted across four dimensions: trend, peak, extreme value, and statistical characteristics (e.g., rising/falling ratios, turning points, and kurtosis). After addressing missing values via median imputation, we removed low-variance features (<0.05) to reduce redundancy and applied Z-score standardization to ensure dimensional consistency. Subsequently, a multi-criteria scoring mechanism (integrating PCA, autocorrelation, and stability) was employed to refine the feature set, ultimately selecting 7 optimal features.

**Figure 3:**
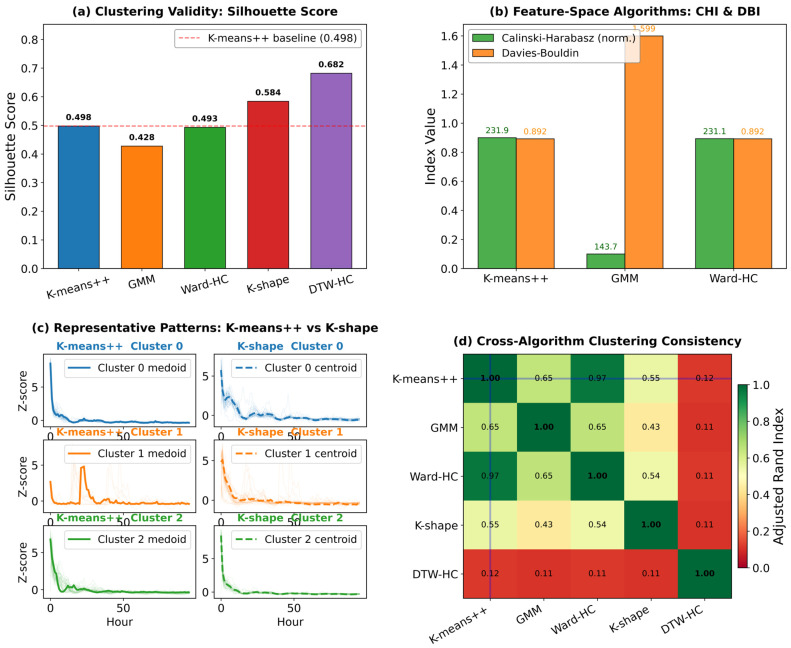
Comparison of time-series clustering algorithms. (**a**) Silhouette scores of five competing algorithms; (**b**) Internal validity indices for feature-space algorithms; (**c**) Representative cluster prototypes derived by K-means++ and K-shape. The light-colored lines in the figure represent the actual comment time series of videos belonging to this cluster; (**d**) Cross-algorithm clustering consistency matrix.

Further, to identify the optimal clustering algorithm, we systematically compared five methods: K-means++, Gaussian Mixture Models (GMM), Ward hierarchical clustering, K-shape, and DTW hierarchical clustering (Figure 3). Based on a comprehensive evaluation of internal validity metrics (Silhouette Coefficient, Calinski–Harabasz Index, Davies–Bouldin Index), cross-algorithm consistency (Adjusted Rand Index), and interpretability, K-means++ was selected as the final clustering algorithm for three primary reasons.

First, it offers an optimal balance between clustering performance and computational efficiency. In the extracted feature space, K-means++ achieved a Silhouette Coefficient of 0.498, slightly outperforming other feature-based alternatives while matching the cluster validity indices of the more computationally intensive Ward hierarchical clustering. Second, K-means++ provides superior semantic interpretability. Unlike shape-based methods (e.g., K-shape) that yield abstract shape averages, the cluster centroids generated by K-means++ directly preserve the peak magnitudes, temporal alignment, and evolutionary trajectories of the original sequences. This geometric fidelity allows for intuitive mapping to real-world dissemination dynamics, such as “early-hour burst” or “afternoon rebound”. Third, the algorithm exhibits high stability within the feature space. The partitions generated by K-means++, GMM, and Ward hierarchical clustering demonstrated strong agreement (ARI ranging from 0.65 to 0.97), confirming a robust, feature-driven underlying structure. Conversely, shape-based algorithms significantly diverged from this consensus (ARI of only 0.12–0.55), indicating that they capture localized waveform fluctuations rather than the broader statistical patterns central to this study.

## 3. Results

### 3.1. Descriptive Statistics

#### 3.1.1. Gender Distribution

**Figure 4:**
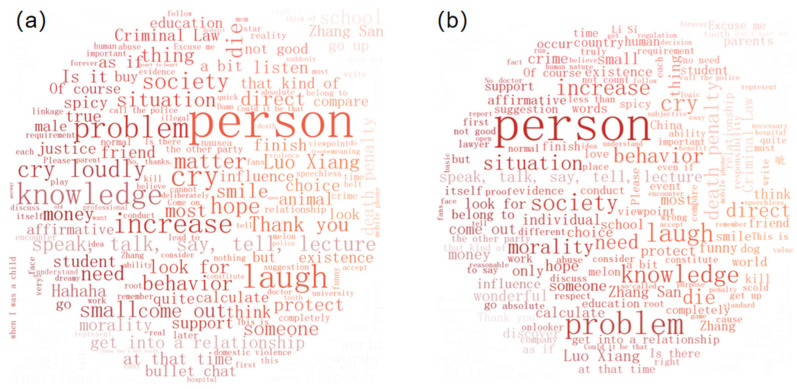
Comparison of high-frequency terms by gender. (**a**) Female users and (**b**) Male users. The top 10 terms for female users include *person*, *laugh*, *cry*, *problem*, *knowledge*, *law*, *haha*, *increase*, *children*, *others*. For male users, the top 10 terms are *person*, *law*, *problem*, *laugh*, *crime*, *knowledge*, *cry*, *society*, *others*, and *increase*.

To investigate gender-specific engagement and comment behavior, we analyzed the audience composition and discourse of the *Luo Xiang Speaks on Criminal Law* account. The results reveal a pronounced gender imbalance: male viewers account for 74.6% of the audience, nearly triple the female representation (25.4%), indicating a higher affinity for criminal law content among men. To further delineate gender-based discussion priorities and linguistic preferences, comment texts were processed separately. Following Chinese word segmentation via the jieba [46] and stop-word filtering, comparative high-frequency word clouds were generated (Figure 4) to visualize these thematic differences.

The textual analysis reveals shared high-frequency terms across genders, including *person*, *law*, *problem*, *knowledge*, *increase*, and *cry*. Notably, *person* emerged as the most frequent term, reflecting a human-centric focus on individual dignity and the protection of rights in specific cases. The consistent prominence of *law*, *problem*, and *knowledge* underscores the audience’s demand for legal literacy and their engagement with complex disputes. Furthermore, the prevalence of *increase* points to a collective aspiration for legal reform, such as intensifying the costs of criminal activity.

Distinct gender-based variations further highlight divergent thematic priorities. Female discourse is characterized by terms such as *child* and *hahaha*; the former indicates a heightened concern for child protection and judicial optimization, while the latter, as an onomatopoeic marker, suggests a tendency to deconstruct legal paradoxes through humor. Additionally, women exhibit higher emotional engagement, with *cry* ranking significantly higher (2nd) than in male comments (6th). Conversely, male discourse prioritizes *crime* and *society*, reflecting a preference for technical analyses of criminal elements and macro-level concerns regarding public order and institutional justice. This suggests that while women engage more affectively, men tend toward systemic and social-impact evaluations of legal cases.

#### 3.1.2. Release Time Distribution

**Figure 5:**
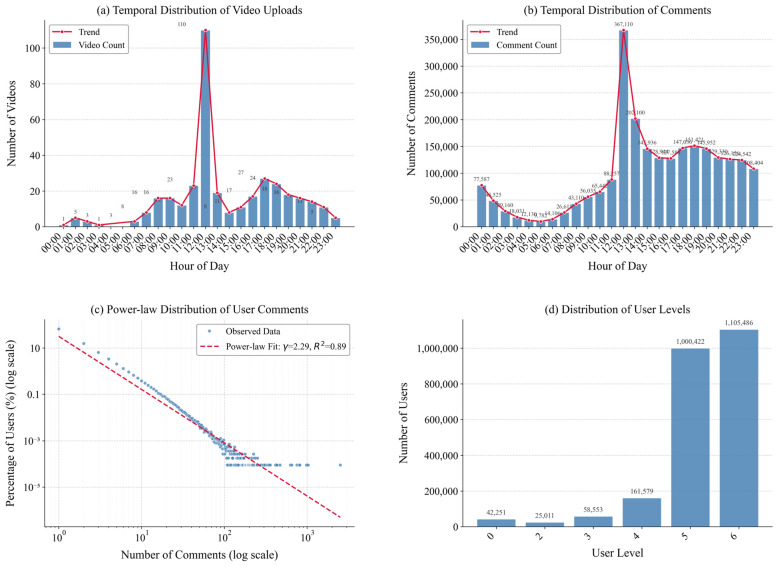
Comment user descriptive analysis. (**a**) Video release time; (**b**) Comment release time; (**c**) Distribution of user comment counts (double logarithmic); (**d**) User grade distribution.

To examine the relationship between video publication schedules and user engagement, we analyzed the temporal distribution of the *Luo Xiang Speaks on Criminal Law* account. As illustrated in Figure 5a, video releases follow a non-uniform bimodal distribution, with high concentrations during the 08:00–14:00 and 16:00–22:00 windows. A distinct peak occurs at 12:00 noon (110 videos), while minimal activity is observed late at night (22:00–07:00) and during the mid-afternoon (14:00–16:00). Such a distribution reflects a deliberate content-posting strategy that mirrors user activity cycles and optimizes daytime productivity. The temporal distribution of user comments also exhibits significant bimodal characteristics, as illustrated in Figure 5b. A major peak occurs at 12:00 (367,110 comments), while a minor peak is observed during the 18:00–19:00 interval (151,421 comments). Engagement significantly diminishes during the late-night to early-morning period (22:00–07:00), reaching its nadir between 03:00 and 06:00. The fact that user activity peaks coincide with video release times indicates a strategic alignment between the creator’s schedule and the audience’s active hours. This synergy ensures that new content is published precisely when users are most likely to interact, effectively optimizing the channel’s reach.

#### 3.1.3. User Engagement Distribution

To evaluate user engagement, this study analyzed the distribution of comment frequencies. As shown in Figure 5c, the results reveal a distinct power-law distribution (γ=2.29), substantiating social network theories where a minority of nodes drive the majority of interactions. Quantitatively, however, the asymmetry is moderate rather than extreme: the Gini coefficient stands at 0.4575, remaining below the threshold of severe concentration (0.50). Specifically, an active minority (7.95% of users) shoulders 39.56% of the total comment volume, within which an elite cohort of less than 0.02% exhibits extreme activity (>100 comments), while the peripheral majority (92.05% of participants) consists of low-engagement users with fewer than 5 comments. This structure balances conversation depth and systemic resilience. Specifically, the active minority drives information diffusion and shapes topical trajectories by cultivating thread depth and reply-chain complexity. Conversely, the substantial volume from the peripheral majority secures the network’s structural resilience against the attrition of individual elite users. Nonetheless, a severe contraction of the active core inevitably flattens conversation trees and diminishes cross-topic fertilization.

#### 3.1.4. Grade Distribution

User levels on bilibili are set from 0 to 6. Based on the active characteristics of bilibili users, level 0 represents newly registered users, whose platform usage is very limited; levels 1 and 2 are classified as inactive users; levels 3 and above are regarded as active users; users at levels 5 and 6 exhibit extremely high interaction frequencies and are considered key users. As shown in Figure 5d, the analysis of user level distribution in this video community shows that the number of users at level 6 is the largest (1,105,486 individuals), followed by users at level 5 (1,000,422). Together, these two tiers account for 88% of all commenting users. This indicates that the community is overwhelmingly dominated by long-term, highly committed key users, whereas lower-level users (level 0~2) exhibit sporadic and discontinuous participation.

#### 3.1.5. Network Structure

**Figure 6:**
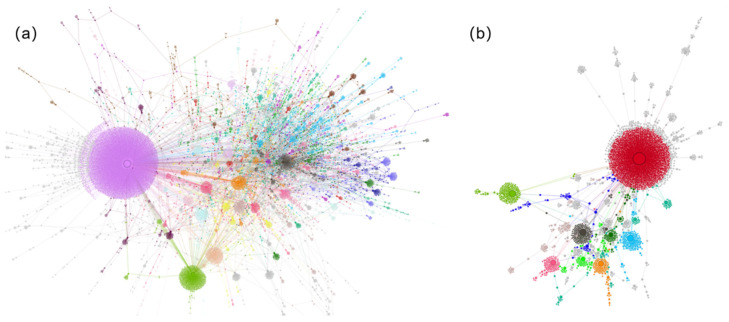
Representative comment networks on bilibili. (**a**) Video: [Luo Xiang] Tencent vs. Lao Gan Ma Dispute—The busy official seal! (Network nodes: 15,808); (**b**) Video: [Luo Xiang] Deceiving machines via system loopholes: theft or fraud? (Network nodes: 3139).

Based on user interactions, 367 directed weighted social networks, denoted as $G=\{G_{i}|i=1,2,\ldots ,367\}$, were constructed (Figure 6). To delineate the social structure and information dissemination paths, we systematically analyzed core topological features, including degree distribution, assortativity, diameter, and connectivity. Results show that 96.5% of the networks have an average degree below 1.0 (mean = 0.6), indicating extreme sparsity where most users form minimal connections. While nearly 30% of nodes are entirely isolated, fewer than 5% exhibit degrees significantly above the mean. This core-periphery architecture suggests that the network relies heavily on a few hub nodes (highly active users) for structural integrity.

Furthermore, the mean assortativity coefficient is −0.19 (standard deviation, σ=0.04), revealing a disassortative mixing pattern. This inherent heterogeneity implies that highly active users predominantly interact with low-activity users, resulting in a top-down, one-way information flow rather than reciprocal engagement. This disassortativity provides a structural explanation for the sporadic, explosive spikes in comment volume observed across specific videos. However, it also highlights an underlying structural deficit: the pronounced lack of horizontal connections among peripheral users inherently limits the formation of localized, diverse sub-communities within the network.

Analysis of network diameters reveals a mean of 25.1, with 92.6% of networks falling below 50. Compared to the typical range of 8~20 in mature social networks [47], these values indicate low-efficiency connectivity and highly linear, non-intersecting interaction paths. Regarding connectivity, 93.7% of the networks exhibit a weak connectivity coverage rate below 60% (mean = 41%), and 84.2% have a strongly connected component proportion of less than 5%. These metrics suggest that users establish transient, indirect links through singular comments, forming fragmented, weakly connected clusters. This structure confirms a model where a few core users provide content while a vast periphery of users consumes without providing feedback [48], resulting in a landscape of isolated small communities.

**Figure 7:**
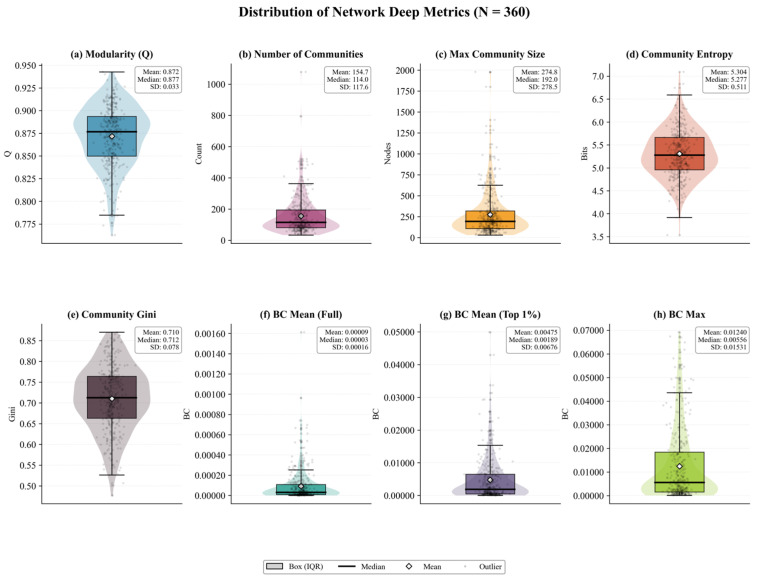
Deep topological metrics of comment interaction networks. (**a**) Modularity; (**b**) Number of communities; (**c**) Maximum community size; (**d**) Community entropy; (**e**) Community-size Gini coefficient; (**f**) The mean betweenness centrality of the entire network; (**g**) Mean betweenness centrality of top 1% bridge nodes; (**h**) Maximum betweenness centrality. The shaded violin body depicts the kernel density estimation (KDE), illustrating the full shape of the empirical distribution beyond the boxplot summary.

To unveil the deep topological architecture of these comment interaction networks, we evaluated their modularity, community size distribution, and centrality features (Figure 7). First, the high mean modularity (0.872) reveals a severely fragmented interaction landscape. Individual comments are largely confined within highly isolated interest clusters, meaning cross-community propagation relies heavily on a limited set of bridge nodes to penetrate these modular barriers. Second, a community-size Gini coefficient of 0.710 with a pronounced right tail indicates extreme scale inequality. Information production is monopolized by a few macro-communities, leaving numerous micro-communities isolated at the periphery; consequently, a user’s information-access opportunity is primarily anchored by their community embedment rather than individual activity levels. Third, the network exhibits a highly polarized betweenness centrality (BC) distribution. While the global mean BC is close to zero, the top 1% of bridge nodes display a mean BC approximately 53 times the network average, capturing the vast majority of shortest paths and acting as critical “structural bottlenecks.”

Crucially, this meso-scale configuration provides the micro-mechanism for the previously observed disassortative mixing: these high-BC bridge nodes anchor the connections between highly active core users and low-activity peripheral users. Their disassortative tie patterns dictate whether interaction intensity can breach community barriers to achieve cross-domain cascade amplification.

### 3.2. Time-Series Clustering Results

#### 3.2.1. Cluster Analysis

**Figure 8:**
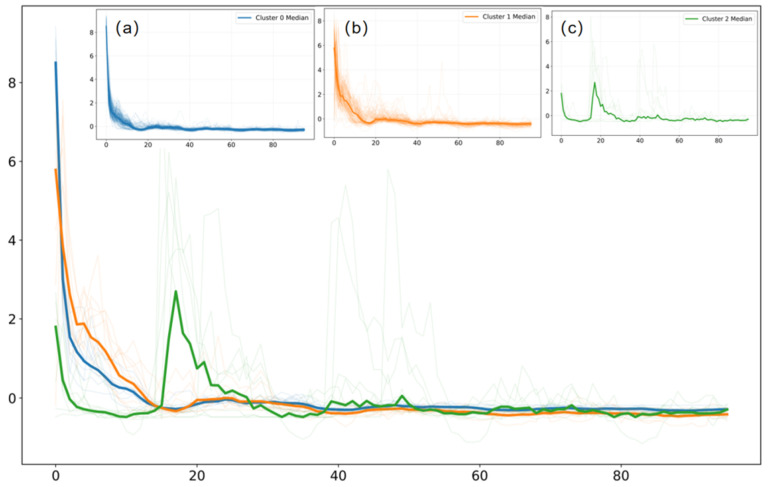
Typical evolutionary patterns of video comments. (**a**) Cluster 0; (**b**) Cluster 1; (**c**) Cluster 2. The light-colored lines in the figure represent the actual comment time series of videos belonging to the corresponding cluster.

To explore the typical temporal evolution patterns of user commenting behavior in video communities and further infer the active lifecycle of videos, this study conducts cluster analysis on the comment time series of individual videos. The results identified three distinct patterns (Figure 8). The first pattern, categorized as burst–decay (Cluster 0), exhibits a primary peak within the first two hours post-release, followed by a sharp attenuation toward a sustained low-level equilibrium with minimal fluctuations. The second pattern, multi-wave oscillation (Cluster 1), similarly demonstrates an initial surge shortly after publication; however, its subsequent decay phase is punctuated by continuous and significant secondary fluctuations. Finally, the delayed peak pattern (Cluster 2) represents a unique non-linear trajectory where, despite substantial initial participation, the true apex of discussion is deferred, emerging only after an interim period of relative quiescence. Collectively, these clusters reveal the diverse engagement dynamics and varying durations of user interest within the video community.

**Figure 9:**
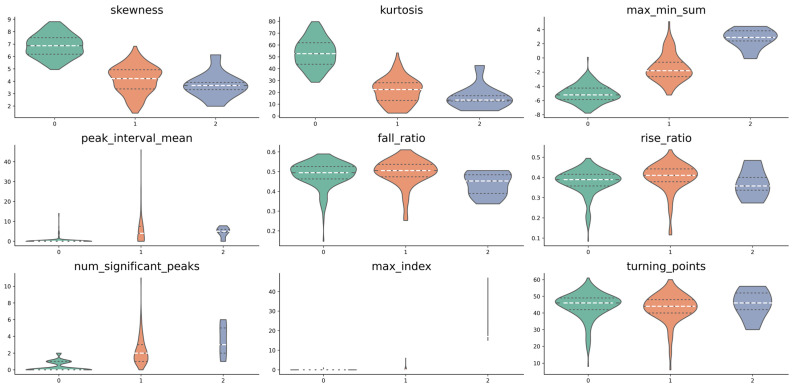
Feature distribution of different clusters. Colors denote cluster identity (green: Cluster 0, orange: Cluster 1, blue: Cluster 2). Within each violin plot, the thick dashed line marks the median, while the two thin dashed lines indicate the first and third quartiles.

The three identified comment evolution patterns exhibit significant disparities across key temporal dimensions, as illustrated in Figure 9. Statistical analysis reveals that four features, differential extremum sum (Max_Min_Sum), maximum index (Max_Index), skewness, and kurtosis, serve as the primary indicators for distinguishing these trajectories. Specifically, Cluster 0 (burst–decay) is characterized by extreme right-skewness and a highly leptokurtic (peaky) distribution, indicating that user engagement is intensely concentrated in the immediate post-release period. In this pattern, the decay rate significantly outpaces the initial growth rate, with minimal subsequent fluctuations. In contrast, Cluster 1 (multi-wave oscillation) displays moderate skewness and kurtosis, reflecting more balanced rise and fall rates and sustained interaction vitality through periodic surges. Finally, Cluster 2 (delayed peak) exhibits the lowest skewness and kurtosis among the three, suggesting a more dispersed temporal distribution. A defining feature of Cluster 2 is its significantly higher Max_Index, as its primary peak is deferred until long after the initial release, whereas peaks for Cluster 0 and Cluster 1 occur almost instantaneously.

From the perspectives of amplitude (max_min_sum, rise_ratio, fall_ratio), distribution concentration (kurtosis,skewness, num_significant_peaks, turning_points), and peak timing (max_Index, peak_interval_mean, rise_ratio, fall_ratio), these clusters represent distinct ecological dynamics within the video community. Cluster 0 manifests as an initial concentrated burst followed by rapid stabilization; the lack of subsequent fluctuations suggests a “flash-in-the-pan” attention model. Cluster 1 demonstrates a “slow-burn” decay punctuated by multiple small-scale oscillations, indicating a more resilient interaction trend where user interest is periodically reignited. Cluster 2 presents a unique “U-shaped” or delayed-growth trajectory; rather than an immediate peak, engagement initially declines or remains stagnant before gradually ascending to a delayed apex. This suggests that user discussion in Cluster 2 is likely triggered by exogenous factors or secondary dissemination in the later stages of the video’s lifecycle, reflecting a latent engagement mechanism that differs fundamentally from the immediate gratification of the burst-type patterns.

#### 3.2.2. Video Attribute Analysis

To further substantiate the distinctiveness of the identified clusters, a comparative analysis was performed on core video attributes, including playback volume, comment counts, likes, and collections. Statistical results (Table 3) indicate that all metrics differ significantly across the three patterns (p<0.001), confirming the validity of the classification. Cluster 0 (burst–decay) is characterized by moderate engagement levels—significantly lower than Cluster 1 but exceeding Cluster 2. In this group, user behavior is predominantly limited to viewing and “light” interactions; while users provide routine feedback through likes or donations, they rarely engage in substantive commentary or proactive sharing. In contrast, Cluster 1 (multi-wave oscillation) yields the highest mean and median values across all participation metrics, suggesting that their content not only attracts a broad audience but also successfully elicits deep interaction and emotional resonance. Finally, Cluster 2 (delayed peak) exhibits the weakest performance, with playback volumes comparable to or lower than Cluster 0 and the lowest median values for all interaction attributes. The dissemination of Cluster 2 content relies heavily on passive recommendation channels or external contingencies, leading to diminished user stickiness and limited sustainability in engagement.

The underlying mechanisms of engagement differentiation across the three patterns can be identified through quantitative contrasts in statistical distributions and network structure. Cluster 1 exhibits moderate *Skewness* (μ=4.13) and *Kurtosis* (μ=21.13), substantially lower than the extreme right-skewness (μ=6.87) and high peakiness (μ=53.21) observed in Cluster 0, yet markedly higher than the low *Skewness* (μ=3.70) and low *Kurtosis* (μ=16.05) characteristic of Cluster 2. This intermediate distributional profile indicates that comment growth in Cluster 1 unfolds as multiple relatively balanced fluctuations along the temporal axis, rather than as a single burst or prolonged dormancy. Furthermore, the significantly lower *Max_Index* of Cluster 1 (μ=0.62)relative to Cluster 2 (μ=25.78) suggests an earlier initial peak, enabling the capture of immediate attention within peak user-activity windows; meanwhile, elevated *Max_Min_Sum* (μ=−1.41) and *Turning_Points* (μ=45) values reveal identifiable secondary fluctuations, signifying sustained participation relay following the initial surge.

At the network level, the multi-wave oscillation morphology of Cluster 1 indicates that hub nodes maintain non-trivial revisit and response behavior after the initial burst, thereby continuously pulling peripheral nodes into interaction through disassortative ties; the rapid post-peak decay in Cluster 0 suggests that hub-periphery connections are transient and lack sustained structural support; the delayed peak of Cluster 2 corresponds to insufficient initial network activation, with later stages dependent on exogenous algorithmic matching by the platform recommendation system.

### 3.3. Predictive Validation of Early-Stage Comment Evolution Patterns

#### 3.3.1. Experimental Design

To support real-time traffic forecasting and proactive resource allocation, we designed a predictive validation experiment to determine if early-stage interaction features can accurately foresee full-lifecycle dynamics. The prediction is formulated as a supervised multi-class classification task using features extracted exclusively from the first 2 h post-release. The target variables are the 96-h full-lifecycle cluster labels established in Section 3.2 (0 = burst–decay, 1 = multi-wave oscillation, 2 = delayed peak). This 2-h window represents the early operational threshold required for platform operators to optimize recommendation weights and bandwidth provisioning before the content lifecycle fully unfolds.

#### 3.3.2. Early-Stage Feature Engineering

To capture the multidimensional signals governing early-stage user engagement, we extract five distinct categories of real-time computable features from the observable data within the first 2 h post-release. These features are designed to map the initial velocity, user demographics, experience structures, textual density, and temporal contexts of the interaction network, as shown in Table 4. Category-specific preprocessing pipelines were constructed to standardize the heterogeneous feature space prior to model training. Continuous numerical variables spanning engagement dynamics, demographics, grade structures, and emotional valence were normalized using standard Z-score transformation (StandardScaler) to mitigate scale-disparity biases. For linguistic analysis, the merged video titles and descriptions were tokenized using the jieba segmenter and transformed into 300-dimensional sparse vectors via TF-IDF encoding. Finally, nominal temporal attributes, specifically release hour, day of the week, and weekend indicators, were converted into a binary feature space using OneHotEncoder, which effectively eliminated unintended geometric ordering among categorical timestamps.

#### 3.3.3. Model Selection and Evaluation

To mitigate model-specific bias, this study systematically compares five classifiers: Logistic Regression, Random Forest, XGBoost, SVM (RBF kernel), and Decision Tree. Given mild class imbalance across the three patterns, SafeSMOTE is integrated into the training pipeline for oversampling, with dynamic k-neighbor adjustment based on the minority-class sample size. The dataset is partitioned via stratified 80/20 split into training and test sets, preserving identical pattern proportions in both subsets. Performance is evaluated by Accuracy, Weighted F1-score, and Macro-AUC to comprehensively assess classification precision, class balance, and ranking capability.

#### 3.3.4. Experimental Results

Table 5 summarizes the predictive performance of the five classifiers. All models substantially exceed the random-guess baseline (Accuracy = 0.333), confirming that interaction data within the first 2 h post-release already encodes identifiable signatures of the final evolution pattern.

Logistic Regression and XGBoost tie for the highest accuracy (0.730), outperforming the random baseline by approximately 40 percentage points. Notably, Logistic Regression achieves the best overall balance across all metrics (Weighted F1 = 0.733, Macro-AUC = 0.859), indicating that the relationship between early-stage features and final pattern labels is approximately linearly separable, and moderate regularization suffices to capture the decision boundary. Its superior AUC implies that the model can reliably rank videos by their probability of belonging to each pattern class, which is critical for operational prioritization.

The predictive feasibility observed above can be understood through the structural correspondence between early signals and lifecycle mechanics. Burst–decay videos typically exhibit extremely high initial growth velocity but low core-user retention, indicating that transient mass attention lacks sustained hub support. Multi-wave oscillation videos display moderate initial velocity with elevated core-user presence, suggesting that hub users are actively seeding the first wave and preparing structural conditions for subsequent revisit-driven participation relays. Delayed-peak videos show low initial velocity and low core-user density, reflecting a “slow-burn” start that relies on delayed algorithmic matching or exogenous social triggers rather than immediate hub mobilization.

## 4. Conclusions

This study focused on the temporal evolution patterns of comment interactions in video communities. Taking 367 videos and 2.39 million comments from the bilibili account *Luo Xiang Speaks on Criminal Law* as an example, it systematically explored the typical patterns and formation mechanisms of comment evolution. The results showed that the audience of *Luo Xiang Speaks on Criminal Law* exhibits significant gender differences (with 74.6% being male), and male users pay more attention to technical issues such as criminal composition and social order, while females focus on topics such as child protection and emotional resonance. Both video publications and user commentary exhibit a synchronized bimodal distribution, with peaks occurring at 12:00 and between 18:00 and 19:00. This strategic alignment suggests that creators’ posting schedules are precisely calibrated to coincide with peak user activity windows, thereby maximizing engagement. Furthermore, comment frequency follows a power-law distribution, characterized by significant participation inequality, i.e., a highly active 10% of the user base contributes 81% of the total discourse. Topological analysis of the interaction networks further confirms a pronounced structural heterogeneity. The vitality of this ecosystem depends heavily on the sustained engagement of a core minority, while interaction patterns reveal a disassortative structure where low-activity peripheral users predominantly gravitate toward and engage with these high-profile hubs.

By establishing a four-dimensional feature system (encompassing trend, peak, extremum, and statistical attributes) and employing multi-criteria feature selection alongside the k-means++ clustering algorithm, this study successfully identified three distinct comment growth patterns with high statistical significance (*p* < 0.001). Specifically, the burst–decay pattern is characterized by a concentrated initial surge followed by rapid quiescence, with the primary peak typically occurring within two hours post-release. In contrast, the multi-wave oscillation pattern features an initial peak followed by a gradual decline punctuated by periodic fluctuations, where its vitality relies on sustained user interaction and secondary dissemination. Moreover, the Delayed peak pattern demonstrates a unique trajectory of initial decline followed by a subsequent rise, with the eventual peak being contingent upon later dissemination opportunities. These three patterns are fundamentally distinguished by key indicators, including the differential extremum sum, maximum index, skewness, and kurtosis, collectively providing a robust framework for understanding heterogeneous engagement dynamics and predicting content lifecycles within video communities. These patterns reveal unique platform-specific dynamics when compared to established literature. Specifically, the burst–decay pattern aligns with the single-peak rapid decay observed on Instagram [49], yet exhibits an accelerated decay rate due to the attention-diversion effects of danmaku and core user guidance. Furthermore, unlike Twitter’s multi-wave propagation that relies strictly on retweets from external opinion leaders [50], the multi-wave oscillation pattern identified here is driven endogenously by platform recommendation algorithms and ongoing event progression.

This study utilized feature-based clustering to identify three distinct evolution patterns within video comment time series. Our findings offer researchers deeper insights into the micro-mechanisms of information dissemination while providing platform operators with a framework for predicting traffic trajectories and optimizing resource allocation based on early-stage engagement. Despite these contributions, the current scope, which is limited to legal-themed content on bilibili, may affect cross-platform and cross-domain generalization. Shaped by its distinctive platform architecture, Bilibili delivers comment growth patterns with markedly stronger nonlinearity and cultural specificity when compared with algorithm-centric short-video platforms. Compared with TikTok or YouTube, Bilibili’s comment evolution patterns constitute a unique coupling of community-driven hierarchical structure and content-depth affordances. Subsequent research will involve comparative analyses across diverse platforms (e.g., YouTube, TikTok) and domains (e.g., entertainment, technology, and lifestyle) to more comprehensively elucidate the complex dynamics of user interaction behavior in video ecosystems.

## Figures and Tables

**Table 1:** Data Description.

Video Information					
bvid	tname	title	descx	view	danmaku
Video ID	Classification	Title	Introduction	View count	Number of bullet comments
reply	favorite	coin	sharex	likex	ctime
Number of comments	Favorited number	Tips received	Number of forwards	Number of likes	Release date
Comment Information					
user_id	user_name	user_sex	user_level	comment	appreciate_count
User ID	User name	User gender	User level	Comment content	Number of likes for the comment
reply_count	user_rpid	user_subject	bv	comment_data	
Number of comment replies	Comment ID	Subject ID	bilibili video ID	Comment time	

**Table 2:** Time series features.

Feature Category	Feature Name	Characteristic Symbol	Formula
Trend characteristics	Percentage increase	*Rise_Ratio*	$Rise\_Ratio=\frac{1}{L_{n}-1}\sum_{i=1}^{L_{n}-1}\delta \left(\Delta x_{i}>0\right)$
Trend characteristics	Percentage of decline	*Fall_Ratio*	$Fall\_Ratio=\frac{1}{L_{n}-1}\sum_{i=1}^{L_{n}-1}\delta \left(\Delta x_{i}<0\right)$
Trend characteristics	Turning point	*Turning_Points*	$Turning\_Points=\sum_{i=1}^{n-2}\delta_{i}$
Trend characteristics	Differential extremum and	*Max_Min_Sum*	$Max\_Min\_Sum=max\left(\Delta x_{i}\right)+min\left(\Delta x_{i}\right)$
Wave peak feature	The number of significant peaks	*Num_Significant_Peaks*	$N_{\mathrm{significant}}=\sum_{i=1}^{M}I\left(x_{i}>H_{final}\wedge \Delta t_{i}\geq D_{min}\wedge P_{i}>P_{min}\right)$
Wave peak feature	Significant peak interval	*Peak_Interval_Mean*	$Peak\_Interval\_Mean=\left\{\begin{array}{lll} \frac{1}{N-1}\sum_{i=1}^{N-1}\Delta t_{i}, & N\geq 2 & \\ 0, & N<2 & \end{array}\right.$
Optimal feature	Maximum distance	*Max_Index*	$Max\_Index=t_{max}-t_{0}$
Statistical characteristics	Skewness	*Skewness (g*_1_ *)*	$\begin{array}{c} g_{1}=\frac{1}{L_{n}}\sum_{i=1}^{L_{n}}x_{i}^{3} \end{array}$
Statistical characteristics	Kurtosis	*Kurtosis (g*_2_)	$g_{2}=\frac{1}{L_{n}}\sum_{i=1}^{L_{n}}x_{i}^{4}-3$

**Table 3:** Analysis of core video attributes.

Characteristics	Overall *p*-Value	Mdn (Cluster 0)	Mdn (Cluster1)	Mdn (Cluster 2)
View (view count)	1.430 × 10^−4^	1687.0k	2269.0k	667.0k
Danmaku (danmaku count)	5.021 × 10^−4^	10.2k	18.1k	7.8k
Reply (reply count)	7.615 × 10^−8^	4.1k	8.0k	2.3k
Favorite (favorite count)	7.929 × 10^−10^	11.0k	27.8k	9.8k
Coin (coin count)	5.181 × 10^−6^	31.0k	59.2k	16.8k
Sharex (share count)	5.634 × 10^−8^	5.1k	11.5k	3.7k
Likex (like count)	1.250 × 10^−3^	151.0k	219.5k	67.2k

Note: Mdn represents the median.

**Table 4:** Prediction features.

Category	Feature Name	Description
Early volume and growth velocity	*T* *wo_hour_comments_per_user*	Proportion of total comments generated within the first 2 h relative to the video’s overall comment volume, capturing initial participation intensity.
	*G* *rowth_percentage_1_2 h*	Ring-ratio growth rate of comment volume from Hour 1 to Hour 2, reflecting the acceleration trend of early engagement.
User demographic structure	*T* *wo_hour_male_ratio*	Male proportion among early commenters (first 2 h).
	*T* *wo_hour_female_ratio*	Female proportion among early commenters (first 2 h).
	*T* *wo_hour_known_gender_ratio*	Proportion of early commenters with disclosed gender information.
	*T* *wo_hour_gender_entropy*	Shannon entropy of gender distribution among early commenters, quantifying demographic diversity.
User grade structure	*T* *wo_hour_level_entropy*	Shannon entropy of user-grade distribution among early commenters, measuring experience-level heterogeneity.
	*T* *wo_hour_high_level_rate*	Proportion of Level-5/6 core users among early commenters, indicating elite-user concentration.
	*T* *wo_hour_low_level_rate*	Proportion of Level-0/2 peripheral users among early commenters, indicating novice-user concentration.
Textual attributes	*C* *ombined_text*	Concatenation of video title (“title”) and description (“descx”); tokenized by jieba and transformed into 300-dimensional TF-IDF vectors (1–2 g, min_df = 2, max_df = 0.8).
	*T* *ext_length*	Character count of the merged title and description, serving as a proxy for content information density.
	*S* *entiment*	Affective valence score computed by SnowNLP, ranging from 0 (negative) to 1 (positive), where values closer to 1 indicate a more positive emotional tone.
Temporal context	*H* *our*	Exact release hour (0–23).
	*D* *ay_of_week*	Release day (0 = Monday to 6 = Sunday).
	*I* *s_weekend*	Binary indicator for weekend release (1 = Saturday or Sunday, 0 = otherwise).

Note: Italics indicate characteristic symbols.

**Table 5:** Prediction results.

Rank	Model	Accuracy	F1_Score	AUC
1	logistic	0.729730	0.732912	0.859382
2	random_forest	0.716216	0.700676	0.844738
3	xgboost	0.729730	0.721258	0.826845
4	svm	0.689189	0.688572	0.800041
5	decision_tree	0.635135	0.626426	0.533477

## Data Availability

Data will be made available on request.
